# Integrating RNA Interference and Nanotechnology: A Transformative Approach in Plant Protection

**DOI:** 10.3390/plants14060977

**Published:** 2025-03-20

**Authors:** Mohammad Shafiqul Islam, Md Robel Ahmed, Muhammad Noman, Zhen Zhang, Jing Wang, Ziqi Lu, Yingying Cai, Temoor Ahmed, Bin Li, Yanli Wang, Abul Khayer Mohammad Golam Sarwar, Jiaoyu Wang

**Affiliations:** 1 State Key Laboratory for Quality and Safety of Agro-Products, Key Laboratory of Agricultural Microbiome of MARA and Zhejiang Province, Key Laboratory of Biotechnology in Plant Protection of MARA and Zhejiang Province, Institute of Plant Protection and Microbiology, Zhejiang Academy of Agricultural Sciences, Hangzhou 310021, China; shafiqmohammadst@gmail.com (M.S.I.); m.noman@zju.edu.cn (M.N.); zhangz@zaas.ac.cn (Z.Z.); wj9311@163.com (J.W.); luziqi18234559092@163.com (Z.L.); 11816053@zju.edu.cn (Y.C.); ylwang88@aliyun.com (Y.W.); 2 State Key Laboratory of Rice Biology and Breeding, Ministry of Agriculture and Rural Affairs Key Laboratory of Molecular Biology of Crop Pathogens and Insect Pests, Zhejiang Key Laboratory of Biology and Ecological Regulation of Crop Pathogens and Insects, Institute of Biotechnology, Zhejiang University, Hangzhou 310058, China; 3 Department of Microbiology, College of Science, King Abdulaziz University, Jeddah 21589, Saudi Arabia; robel.zstu@gmail.com; 4 Xianghu Laboratory, Hangzhou 311231, China; temoorahmed@zju.edu.cn; 5 Department of Life Sciences, Western Caspian University, Baku 1001, Azerbaijan; 6 Laboratory of Plant Systematics, Department of Crop Botany, Bangladesh Agricultural University, Mymensingh 2202, Bangladesh; drsarwar@bau.edu.bd

**Keywords:** fungal disease, nanotechnology, plant protection, plant–pathogen interactions, RNAi

## Abstract

RNA interference (RNAi) has emerged as a potent mechanism for combating pathogenic fungi and oomycetes over the past decades. It offers a promising gene-silencing approach by targeting crucial genes involved in diseases caused by economically and scientifically significant fungal pathogens, such as *Botrytis cinerea* and *Fusarium* species. Simultaneously, nano-agro-products have gained attention as alternatives to traditional fungicides in plant protection strategies. However, the instability of naked RNA molecules outside the cellular environment presents a challenge, as they degrade rapidly, limiting their efficacy for prolonged disease control. Concerns regarding the toxicity of protective nanoparticles to non-target organisms have also arisen. Integrating RNAi with nano-agro-products, particularly nanocarriers, to form RNA-nano complexes has demonstrated significant potential, providing enhanced RNA stability, reduced toxicity, and extended disease control. This review explores the mechanisms of RNA-nano complexes-mediated plant protection, addressing RNA stability and nano-toxicity issues while examining the prospects of RNA-nano complex research in plant pathogen management.

## 1. Introduction

Fungal diseases cause significant losses in agriculture, which are estimated to be about 20~30% of the global crop yield. Sometimes, fungal diseases exacerbate the economic hardships, posing threats to global food security. The impact of fungal epidemics is severe in many regions of the world, especially in regions (including Asia) where agriculture is a major contributor to GDP [1,2]. Besides the loss in overall production level, some fungal pathogens are responsible for food toxicity and produce harmful mycotoxins. Such toxins can easily enter the food chain, posing a significant threat to food safety [3,4]. For example, *Fusarium graminearum* is a fungal pathogen responsible for causing Fusarium head blight disease in cereal crops. This pathogen produces a mycotoxin called deoxynivalenol (DON), which has adverse health effects on humans and animals [5,6]. Addressing fungal diseases requires integrated management strategies, including resistant crop varieties, chemically synthesized fungicides [7], biological controls, and robust global surveillance systems, to mitigate their effects on agriculture and food security [3,8].

However, extensive use of chemical fungicides and inefficient biological measures have become impractical due to their off-target effects and eco-safety issues, necessitating the development of innovative control measures to sustain agricultural productivity and minimize crop losses [9]. Therefore, efforts are being made to formulate different types of RNA-based bio-fungicides as an alternative solution [10]. Various types of RNA regulate gene-silencing to protect crops from pathogen attacks. Gene-silencing is an influential technique for controlling gene function and preventing many fungal plant diseases [11,12]. This gene-silencing method significantly impacts numerous biological processes, particularly innate immunity in plants [13]. RNA interference (RNAi) approaches offer a promising means of safeguarding host plants from pathogenic infection through potent RNAi signals produced within the plant, a technique known as host-induced gene silencing (HIGS) [14]. Transgenic plants using HIGS produce genetically modified foods (GMOs), which remain controversial in society. However, certain constraints, including the lack of a stable genetic transformation system for many economically significant crops, along with the high costs of developing, registering, and maintaining GMO crops, as well as public acceptance challenges, hinder the feasibility of using HIGS as a disease management strategy against fungal infections [10,14].

**Figure 1:**
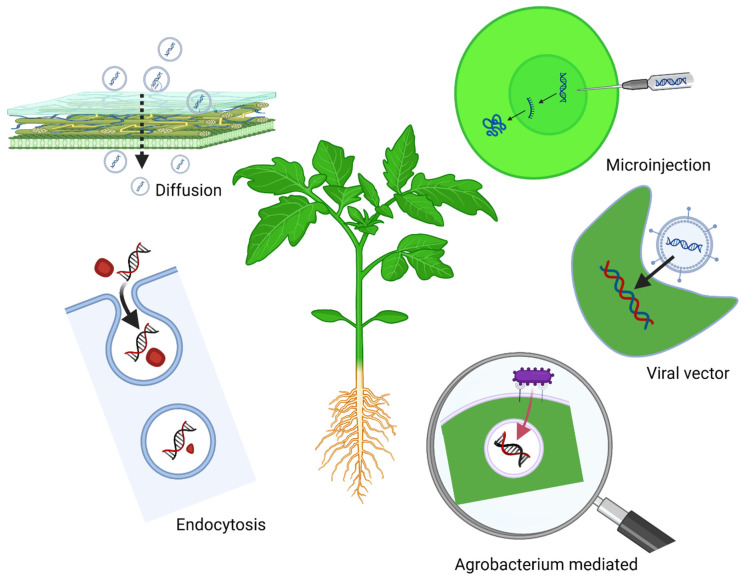
Different nucleic acid delivery methods in plants for RNA interference. Various approaches are used to deliver molecules into cells. Diffusion: small molecules pass through cell layers. Endocytosis: molecules pass through cellular vehicles into the target site. Microinjection: molecules are directly injected into the cellular cytoplasm. Viral vectors: these utilize plant viruses to deliver genetic molecules into plant cells. Agrobacterium-mediated: *Agrobacterium tumefaciens* ensure the gene transfer inside plant cells. Figure created with Biorender “https://app.biorender.com/user/signin (accessed on 18 February 2024)”.

RNAi-based strategies can control agronomically important fungal pathogens by delivering dsRNA through spraying or by injecting into plants (Figure 1). However, successful application requires optimizing dsRNA design, improving delivery methods, and ensuring that target fungi possess functional RNAi machinery and the ability to uptake external RNA [15]. An innovative approach known as spray-induced gene silencing (SIGS) has proven effective in controlling *Botrytis cinerea* by spraying exogenous double-stranded RNA (dsRNA), small interfering RNA (siRNA), and hairpin RNA (hp-RNA), which trigger post-transcriptional gene-silencing via RNAi [16]. SIGS protects pre-harvest and post-harvest crops from fungal pathogens by ensuring high RNA uptake efficiency [17,18]. Therefore, SIGS RNAs targeting genes associated with RNAi machinery and siRNA biogenesis have demonstrated significant success, making these biological pathways promising targets in other organisms [19]. One major drawback of SIGS is the inherent instability of RNA in external environments, particularly under conditions of high temperature, humidity, or ultraviolet radiation exposure [19,20,21,22].

Recently, nano-enabled approaches have been devised to ensure safe and stable delivery of RNA molecules into plants [23]. This technology uses nanocarriers of various sizes and shapes, ensuring the targeted delivery of RNA molecules into specific cells or tissues. This technology has been effectively used in plant protection against potato late blight disease [24]. Additionally, nanoparticle-coated siRNA increases the effectiveness of SIGS-mediated gene silencing. For example, functionalized carbon dots (CDs) are employed to form complexes with the selected dsRNAs (dsRNA-CDs) through electrostatic interactions and effectively controlled *Phytophthora* infection in potato [25]. Another alternative is a nanotube-based system, which can be directly used to deliver RNA material into intact plant cells [26]. This review will explore the current scenario of RNAi, as well as its integration with nano-agro-products for plant protection, drawing conclusions and providing insights for future research, with a focus on the use of nano-based approaches in sustainable plant protection with minimal/no environmental damage.

## 2. The Role of RNAi in Advancing Crop Protection Strategies

RNAi usually regulates various biological processes by interfering with mRNA translation. MicroRNAs (miRNAs) and small interfering RNAs (siRNAs) are distinct classes of small non-coding RNAs that play key roles in RNA interference (RNAi)-mediated post-transcriptional gene regulation. Despite their shared involvement in gene silencing, they differ in their origins, biological functions, and mechanisms of action [27]. miRNA is produced endogenously from their gene, whereas siRNA can originate from exogenous (e.g., viral infections, artificial introduction) or endogenous sources, such as dsRNA or transposons. These two types of RNA molecules are used significantly in RNA silencing to protect plants from pathogen infection [28].

**Figure 2:**
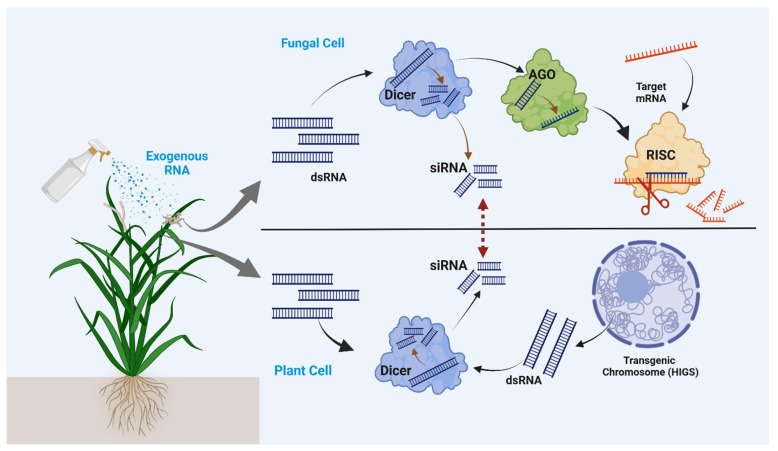
Step-by-step exogenous dsRNA or sRNA-mediated gene silencing for crop protection against pathogens. The arrow sign between fungal and plant cells shows the cross-kingdom transfer of potential siRNA molecules produced from transgenic plants. Exogenously applied dsRNA material is primarily processed by hosts Dicer and AGO proteins, which are finally incorporated into RISC complex for gene silencing. Figure created with Biorender “https://app.biorender.com/user/signin (accessed on 19 January 2024)”.

Research suggests that miRNAs play a crucial role in the biological stress response of plants [29] and have protective roles in plant diseases induced by harmful bacteria and fungi. The first plant endogenous miRNA involved in plant biological stress is mi-ATGB2, which controls ETI (effector-triggered immunity) associated with R gene expression [30], the second layer of plant immune defense. As mentioned earlier, siRNAs can be synthesized and externally applied to plants, so siRNA is generally considered more effective against fungal and other pathogens compared to naturally occurring miRNA. After entering the cell, long dsRNA molecules undergo processing by the DICER and are converted to small 20–25 nucleotide siRNA. siRNA is subsequently incorporated into the RNA-induced silencing complex (RISC) (Figure 2), the most crucial center of siRNA-facilitated gene silencing. A single strand of the siRNA, referred to as the guide strand, guides RISC to target mRNA sequences with complementary bases [31]. This interaction leads to the breakdown of the target mRNA, effectively silencing gene expression. During the spray or foliar application of RNA-mediated gene silencing in crop protection, siRNA targets the specific gene (for example, *DCL 1*, *DCL 2*) and plays a significant role in disease control [32].

## 3. Mechanisms of Exogenous RNA Uptake by Crop Plants and Pathogens

Plants use several key mechanisms to uptake exogenous RNA molecules, which is the most crucial step in gene silencing and pest management [33,34,35]. Based on the uptake mechanism, they can be categorized into direct and indirect methods. Direct uptake can be performed by cellular uptake or endocytosis. The cellular uptake process can be performed directly through leaves or stems. In this case, molecules diffuse through the plant cuticle and cell walls. After entering the cells, dsRNA molecules go through extensive processing before interacting with silencing machinery (Figure 2). During this period, several environmental and cellular factors affect the effectiveness of the administered RNA [36]. Endocytosis is another mode of RNA uptake in plants controlled by intracellular vesicles. These vesicles are used to transport the RNA material into the cytoplasm and active site for RNA silencing, such as RNA-induced silencing complexes (RISC) [37]. Some studies on endocytosis reported that specific receptors on the cell membrane, pH, and temperature have a major impact in plants during this process [38].

On the other hand, indirect exogenous RNA uptake can be performed by root uptake, microinjection, viral vector system, *A. tumefaciens*-mediated transformation, and nanoparticle delivery by passive diffusion. Root uptake is facilitated by passive diffusion or active transport across root membranes. The absorption efficiency is increased by using carriers or protectants [32]. The micro-injection technique allows for tailored distribution by directly injecting RNA into particular plant tissues or cells. Although accurate, it requires much work and is not as practical for large-scale applications. RNA molecules can also be introduced in plants by engineered viral vectors. Viruses effectively silence genes using their innate capacity to infect cells and distribute RNA [39]. In addition, *Agrobacterium tumefaciens* are widely used in genetic engineering to deliver genetic material to plants. It also applies to targeted gene silencing applications by delivering associated cDNA molecules [40]. Moreover, nanotechnology offers advanced methods for RNA delivery by enhancing RNA stability and facilitating its transport across plant membranes via passive diffusion or receptor-mediated pathways. It also minimizes degradation and ensures controlled release of RNA material.

## 4. Mechanism of RNAi-Based Crop Protection in Plants

RNAi-based plant protection against plant pathogens mostly depends on the uptake and processing of dsRNA or siRNA molecules by fungal hyphae or plants, which can be directly internalized from the environment or indirectly in the transgenic plant (Figure 2). Recent studies provide evidence for the uptake of dsRNA and siRNAs, referred to as ‘environmental RNAi’, by fungal pathogens, which can trigger gene silencing within fungal cells. This mechanism enables the direct or foliar application of pathogen-targeting RNAs onto crops, thereby silencing fungal virulence genes to enhance plant protection [18]. While fungal cells can successfully take up RNA molecules, the RNAi machinery is essential for efficiently silencing target genes [15]. After plant cells uptake the siRNA molecules, they transfer the processed and activated siRNA to the pathogenic cells, which are further utilized to silence pathogenic genes. RNA-binding proteins, like *AGO* and *DCL*, facilitate the processing of dsRNA into siRNA, which guides gene silencing via the RISC, as mentioned earlier. The current study supports different types of siRNAs capable of traveling between organisms and hosts, called cross-kingdom RNAi [41]. Cross-kingdom trafficking of siRNA disperses the RNAi information and may have a significant role in community resistance against specific pathogenic agents. Cross-kingdom RNAi involves multiple siRNA translocation mechanisms. Some studies suggest that siRNAs are transferred as internal cargo within extracellular vesicles (EVs), as seen in *Arabidopsis*, where EVs deliver siRNAs to *Botrytis cinerea* for virulence gene silencing [42]. Conversely, other reports indicate that a significant portion of siRNAs in *Arabidopsis* apoplastic fluid exist outside EVs, stabilized by RNA-binding proteins such as ARGONAUTE2 (AGO2) and GLYCINE-RICH RNA-BINDING PROTEIN 7 (GRP7) [43]. These findings highlight the complexity of siRNA transfer, with potential differences in stability and efficiency. Given the regulatory challenges of transgenic crops, environmental RNAi via direct dsRNA application offers a promising alternative for controlling fungal, insect, and viral pathogens [37,44,45].

A major challenge in siRNA delivery is the instability of RNA before uptake by fungal pathogens or plants [46]. To enhance RNA stability and uptake, researchers have developed strategies, such as nanocarrier-based delivery systems and chemical stabilization of dsRNA. These advancements enable efficient RNA uptake, providing a targeted and eco-friendly strategy for controlling agricultural pests and pathogens.

## 5. Nano-Enabled Delivery Systems for dsRNA/siRNA Stabilization

Nano-enabled RNA delivery systems in plants are designed to enhance the stability, uptake, and efficacy of RNA molecules [47]. Nanocarrier-based strategies, such as encapsulating dsRNA in liposomes or chitosan nanoparticles, protect dsRNA from UV radiation and RNase activity, improving delivery efficiency (Table 1) [21]. Furthermore, terminal modifications prevent exonuclease degradation, such as capping dsRNA’s 5′ or 3′ ends. These advancements have facilitated the widespread adoption of RNAi and nanotechnologies, particularly SIGS (Table 2), to manage agricultural pests and pathogens [48].

**Figure 3:**
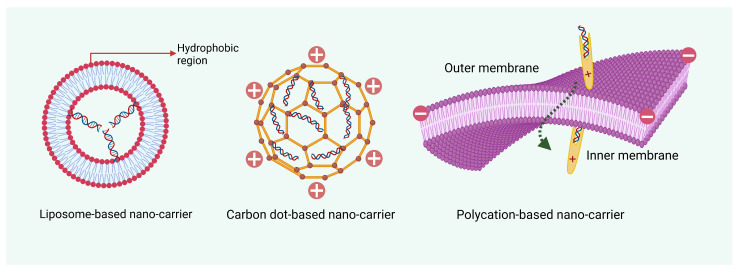
Various types of nano-enabled nucleic acid carriers. These carriers ensure the efficient delivery of nucleic acid in the target cell without damage to environmental or cellular factors. Figure created with Biorender “https://app.biorender.com/user/signin (accessed on 20 January 2024)”.

Additionally, various chemical modifications have been developed to overcome the challenges of nucleic acid instability during delivery to plants [49] (Table 1 and Table 3). However, while utilizing these systems, several key factors must be considered. The delivery system should protect RNA from degradation, enhance RNA uptake into plant cells, and allow for controlled release to ensure effective gene silencing. Additionally, the materials used must be safe and compatible with plant tissues. These advanced nano-enabled systems represent a cutting-edge approach in plant biotechnology, offering significant potential for enhanced gene regulation, pest management, and crop protection (Figure 3).

**Table 1:** Nano-enabled delivery methods and their applications in plants and animals.

Name	Composition	Function	References
Liposomes	The lipid bilayer is a vesicle that encapsulates and transports the RNA molecules.	Enhanced stability can be engineered to use nanoparticles in a controlled manner.	[50]
Metal nanoparticles, like gold or silica	Bind with RNA molecules and carry them into plant cells through endocytosis.	Stability enhancement	[34]
Polymeric biodegradable nanoparticles	Polylactic-co-glycolic acid (PLGA) and chitosan can form nanoparticles that encapsulate RNA molecules.	Provide stability to RNA and release RNA inside in a controlled manner.	[51]
Carbon nanotubes	Cylindrical nanostructures with colossal surface area.	Enormous surface area, it can be used to carry significant amounts of RNA.	[25]
Exosomes and nanovesicles	Naturally occurring nanoparticles derived from cells.	It can be loaded with RNA and used to deliver it to plant cells, having natural compatibility.	[52]
Nanospheres and nanorods	Spherical or rod-shaped nanoparticles made from various materials like metals, polymers, or silica.	Designed to deliver RNA molecules by attaching them to their surface or incorporating them into their structure.	[53]
Dendrimers	Dendrimers are highly branched, tree-like polymers.	Used to deliver RNA molecules by encapsulating them within their structure.	[54]
Micelles	Micelles are self-assembled nanoparticles formed from amphiphilic surfactants.	Can encapsulate RNA molecules in their core and facilitate their uptake into plant cells.	[55,56]
Nucleic acid nanostructures	RNA molecules can be assembled into nanostructures, like RNA nanorods or RNA nanoparticles.	These structures can improve the stability and cellular uptake of RNA molecules.	[57]
Magnetic nanoparticles	Magnetic nanoparticles produced from materials like iron oxide.	Employed to direct the delivery of RNA molecules to plant tissues through the application of an external magnetic field.	[58]

**Table 2:** Spray-induced gene silencing (SIGS) approaches to control plant pathogens.

Pathogen	Host	Spray RNA	Target Gene	Main Effect	Reference
*Magnaporthe oryzae*/ Rice blast	Rice	dsRNA	MoDES11	Systemic disease inhibition	[59]
*Fusarium graminicum*	Barley	dsRNA	CYP51-A, CYP51-B, CYP51-C	Restricts the growth of necrotrophic fungus	[60]
*Fusarium graminicum*	Barley	dsRNA	FgAG-01, FgAG-02	Containment of infection areas	[61]
*Fusarium graminicum*	Barley	dsRNA	FgDCL1, FgDCL2	Reduced fungal infection	[61]
*Fusarium graminicum*	Wheat	dsRNA	Myosin5	Reduction in phenamacril resistance	[62]
*Botrytis cinerea*	Tomato, strawberry, grape, lettuce, onion, Arabidopsis, Grapevine	dsRNA	Bc-DCL1/2	Inhibition of fungal growth reduced disease symptoms and suppressed fungal transcripts. BcCYP51, Bcchs1, BcEF2	[32]
*Phytophthora infestans*/ Potato Late Blight	Potato	dsRNA	SDH, EF-1a, GPI, HAM344, PLD-3, HSP-90	Enhanced disease resistance and less sporulation	[63]
*Phytophthora infestans*/ Potato Late Blight	Potato	dsRNA	PiGPB1, Pihmp1, PiCut3, PiEndo3	Reduction in disease progression	[64]
*Plasmopara viticola*/ Grapevine downy mildew	Grapevine	dsRNA	PvDCL1/2	Reduced disease progress rate	[65]
*Phakopsara pachyrhizi/* Soybean rust	Soyabean	dsRNA	ATC, GCS-H, RP-S16	Reduction in fungal biomass and a lower number of pustules on leaves	[66]
*Rhizoctonia solani*	Rice	dsRNA	DCTN1 + SAC1, PG	Transport of vesicles, pectin degradation	[67]

**Table 3:** Existing exogenous RNA delivery methods and their application in plants.

Delivery Vehicle	Delivery Mode	Target Site of Pathogen	Exposure	Durability	Efficacy	Reference
LDH	*A. thaliana* leaves or spray atomizer on *V. unguiculata* and *N. tabacum*	Silences the PMMoV replicase gene and the CMV target gene.	200 µL samples of 15 µg CMV2b-dsRNA–LDH, sprayed on day 0 only.	After 2 min, uncoated (dsRNAs) showed minimal degradation, but dsRNA-loaded layered double hydroxides (dsRNA-LDHs) remained functionally intact.	Days 1–5 application: LDH-only treated plants developed more necrotic lesions than dsRNA-LDH-treated plants. LDH-dsRNA provided superior protection against the virus 20 days after application. Disease severity includes 10% for leaf spray, 15% for petioles adsorption, and 35% for sinking roots.	[68]
LDH	Spray the leaves, immerse the petioles, or drip the roots	The transcriptional activity of the fungus gene FoCYP51.	Spray leaves and petioles with 300 µg dsRNAs in 3 mL ddH_2_O per plant.	Degradation of naked dsRNA lasted from 1 to 10 min.		[69]
CD (Carbon Dots)	Low-pressure spray	GFP transgene and endogenous gene silencing in *N. benthamiana* and *S. lycopersicum*.	SiRNA/CD is sprayed on plants at 12 ng/µL concentration on Days 1, 7, and 14.	SiRNA/CD is sprayed on plants at 12 ng/µL concentration on Days 1, 7, and 14.	Naked dsRNAs degrade entirely after 15 min. The dsRNA-CDs remain intact after 60 min of incubation.	[70]
	Exogenous Spray	Fungus sporangia, CDQ complex.			Naked dsRNA does not show an effect, but dsRNA CQDs mixture 10 µL/mL shows a significant inhibitor.	[24]
CD bPEI-CD branched polyethylenimine	Petiole absorption and leaf spray	Virus. RNA polymerase and coat protein genes of grapevine leafroll associated virus-3 silenced.	The 0.00092 g/mL concentration was diluted 32 times.	Naked dsRNAs degrade after 2 h, while dsRNA-CDs-bPEI remains intact.	After a single dose, the virus titer dropped over three weeks, but several doses are necessary to enhance fruit quality.	[71]
CNT (Carbon nanotube)	Infiltration of *N. benthamiana* leaves using needleless syringes	The host plant silences mGFP5 transgenes in leaves.	100 nM siRNA, 2 mg/L SWNT.	The degradation of naked dsRNAs was 94% after 6 h. 30% degradation of dsRNA-SNWT after 6 h.	Gene silencing effectiveness reached 95% within 1 day of invasion.	[25]
CPP (Cell-penetrating peptide)	Applying needleless syringe infiltration to *A. thaliana* leaves	For insects, silence GFP and firefly luciferase genes.	For up to 36 h, incubate 100 µL of dsRNA-peptide.	After 12 h, naked dsRNAs showed minor degradation, but dsRNA-peptides remained intact.	Naked dsRNAs did not show silencing effects, whereas dsRNA-peptides showed genetic down-regulation within 12 to 36 h.	[72]
Gold Nanoparticle	Various insect cell lines	Silences Spodopteria, frugiperda luciferase gene.	dsRNA (50 µg/mL)	Endosomal escape was improved by dsRNA-Au compared to dsRNA alone.	Luciferase activity for dsRNA-Au is reduced by up to 58% compared to dsRNA alone.	[73]
	Needleless syringe infiltration *N. benthamiana* leaves	Inhibits mGFP5 transgenes in *N. benthamiana* leaves.	100 ng siRNA	The degradation of naked dsRNAs was concluded after 30 min, while the dsRNA-Gold NP remained intact.	No NbrbohB overexpression indicates minimal stress to plant tissues.	[74]
Chitosan Nanoparticle	Not tested	Silences tomato mosaic virus CP gene.	Use 200 µg/mL of dsRNA-chitosan.	Not reported	Low toxicity and no inhibition of root development are seen with dsRNA-chitosan.	[75]
SPc (star polycation)	To treat *Myzus persicae*-infested oilseed rape leaves, use a pneumatic water sprayer	Silences genes (ATP-A: 413 bp, LOC111039523; ATP-d: 383 bp, LOC111041166; ATP-G: 301 bp, LOC111040044) of *M. persica.*	Spray 0.2 µL dsRNA/SPc formulation on Day 0.	After 1.5 h, naked dsRNAs completely degraded in 12.5% of aphid hemolymph, though dsRNA-SPc remained intact.	Control effectiveness was 61% on Day 3 and 50% till Day 6 after SPc-dsRNA treatment.	[76]
	Drenching roots	Silence genes associated with wing formation in *M. persicae*, such as vg and Ubx.	Applying 200 µL dsRNA/SPc formulation to radish seedlings on Day 0 before transplanting with *M. persica*.	Not reported	Approximately 40% of *M. persica* grew functional wings using both dsRNA genes.	[77]

## 6. Nanotechnology-Based Enhanced Crop Protection Against Pathogens

Integrating nanotechnology with RNA interference (RNAi) offers a groundbreaking approach to managing crop diseases by targeting pathogens at the molecular level. Nano-RNAi systems employ nanomaterials as carriers to deliver RNA molecules, such as small interfering RNA (siRNA) or double-stranded RNA (dsRNA), to plant cells or directly to pathogens [78]. These nano-systems enhance the stability, delivery, and uptake of RNAi molecules, overcoming key limitations of conventional RNAi delivery methods [79]. Nanocarriers, including liposomes, dendrimers, and polymeric nanoparticles, protect RNA molecules from enzymatic degradation and ensure their targeted delivery [80]. Upon uptake by the plant or pathogen, these RNA molecules silence essential pathogen genes through sequence-specific degradation of messenger RNA (mRNA), disrupting vital processes in the pathogen lifecycle. For instance, nano-RNAi systems have been shown to effectively suppress fungal infections by targeting chitin synthase genes in fungi or viral replication genes in plant viruses [81]. Not only are nanoparticles effective against pathogenic fungi, but they also exhibit efficacy against bacterial infections. For instance, copper oxide nanoparticles (CuO-NPs) have been shown to combat bacterial leaf blight disease in rice [82].

Nanotechnology systems offer unparalleled precision and environmental sustainability compared to traditional chemical pesticides. The RNAi-based mechanism of pest control minimizes the off-target effect and reduces the use of agrochemicals. It ensures the survival of nonpathogenic organisms, which is beneficial to the crop and a prerequisite for sustainable agriculture [83,84]. However, there are several barriers and challenges, such as feasibility for agricultural use or production cost, and approval from regulatory bodies always remains for new technologies. More precisely, in vitro dsRNA production for small-scale use and high purity costs approximately USD 60/g, whereas large-scale production using advanced microbial fermentation methods reduces the cost to around USD 2/g after successful field trials [85]. Further optimization by some companies has brought production costs down to approximately USD 1/g [85]. However, RNAi-based biopesticides remain more expensive than traditional chemical pesticides, highlighting the need for industry involvement and innovative approaches to improve cost-effectiveness for agricultural applications [86]. RNAi-based plant protection products face a complex and evolving regulatory landscape that varies by country. In the USA, they are classified as biopesticides under Code 40 of Federal Regulations (CFR) Part 158 and are not considered GMOs unless they involve transgenic bacteria producing dsRNA [87,88]. In Australia, dsRNA-based products are regulated as agricultural chemical products by the Australian Pesticides and Veterinary Medicines Authority (APVMA) and are exempt from GMO classification if the dsRNA does not undergo translation [87]. The European Union classifies HIGS-based products as GMOs, whereas SIGS-based products are regulated for crop protection [87,88]. Regulatory approval also requires assessment of product formulation, biocompatibility, non-target effects, and environmental impact to ensure safety and efficacy.

Despite these challenges, advancements in nanotechnology and RNAi synthesis continue to expand the scope of this innovative approach [89]. In recent years, the use of microbe-derived nanoparticles (NPs) as nano-pesticides has gained significant attention among cultivators, particularly for managing plant diseases [85,90]. This emerging approach is increasingly favored over conventional physical and chemical methods due to its potential benefits. Nano-RNAi systems represent a promising frontier in crop protection, offering a sustainable and precise method to combat diverse plant pathogens while safeguarding the environment and ensuring food security.

## 7. Concluding Remarks and Future Directions

Ongoing research on nano-enabled delivery strategies has raised new promises in the field of plant protection and agriculture. As we know that plant systems have their own mechanisms to defend against nature or invaders, like pathogens, such mechanisms make it difficult to insert outer materials inside the cell. While designing the nanoparticle-based delivery system in plants, such complex or other cellular structures need to be considered. Sometimes it is very hard to deliver specific nanoparticles to the specific plant tissue. In that case, the nanoparticles having higher specificity and accuracy should be considered. Exogenous nucleic acids, especially RNA material, are more vulnerable and prone to degradation by the natural environment or plant system. Therefore, encapsulation of the RNA molecules for delivery into the plants can ensure the success of the goal [80,91]. The uptake and localization of exogenously applied dsRNA in plants critically influence its processing and RNAi efficacy. If dsRNA enters the symplast, it is processed by plant dicer-like enzymes (DCLs) into siRNAs, which may not possess the optimal properties for effective RNAi in target pests or pathogens [92]. In contrast, dsRNA that remains in the apoplast avoids plant DCL processing and can be directly taken up by fungal pathogens, where fungal DCLs generate siRNAs for gene silencing. Therefore, apoplastic localization of dsRNA is advantageous when targeting pests or pathogens [93]. Supporting this, transplastomic plants expressing unprocessed dsRNA have shown greater pest control efficacy than transgenic plants producing siRNAs, highlighting the importance of dsRNA stability and localization in RNAi-based crop protection.

There is an important factor in the fate of delivered nanoparticles in the environment, especially in soil or water systems. Studies have reported that certain chemically synthesized nanocarriers used for RNA-based pesticide delivery can induce toxicity in non-target organisms and pose risks to the food chain [94]. These findings underscore the need for biodegradable and biocompatible nanocarriers to ensure the safe and sustainable application of nanotechnology in agriculture. On the other hand, the existing mechanism of nucleic acid delivery to mature plants or specific tissues are less efficient. Improvements in CRISPR/Cas systems or RNAi technologies integrated with nanoparticle delivery could enhance gene editing efficiency for improved plant protection. While nano-enabled delivery methods have shown promise in laboratory experiments, scaling up these methods for large-scale agricultural applications is still challenging, especially considering nanoparticle production cost and complexity. Developing cost-effective, large-scale synthesis methods for nanoparticles is essential for the commercial viability of these technologies.
